# Characterization and Nutritional Intervention Effects of *Canna edulis* Type 5 Resistant Starch in Hyperlipidemia Mice

**DOI:** 10.3390/foods14010092

**Published:** 2025-01-02

**Authors:** Houxier Li, Nan Wang, Jiahui Wu, Shuting Tan, Yan Li, Nan Zhang, Li Yang, Aji Li, Rongting Min, Maochun Xiao, Shiyao Su, Xiang Wang, Xueyong Wang

**Affiliations:** 1 School of Chinese Meteria Medica, Beijing University of Chinese Medicine, Northeast Corner of the Intersection of Sunshine South Street and Baiyang East Road, Fang-Shan District, Beijing 102488, China; 15928912991@163.com (H.L.); wujiahui2017@126.com (J.W.); tstyuki@163.com (S.T.); liyan9696966@163.com (Y.L.); maomao1112023@163.com (N.Z.); yl220221@163.com (L.Y.); laj2243303997@163.com (A.L.); 15807919980@163.com (R.M.); 13036677850@163.com (M.X.); 13555737250@163.com (S.S.); wangxiang120312@163.com (X.W.); 2 Air Force Medical Center, No. 30 Fucheng Road, Hai-Dian District, Beijing 100080, China; 18811385728@163.com

**Keywords:** type 5 resistant starch, hyperlipidemia, gut microbiota

## Abstract

Numerous reports have indicated that the type 3 resistant starch (RS3) derived from *Canna edulis* can regulate lipid metabolism. However, it remains unclear whether the type 5 resistant starch (RS5) exhibits similar effects. In this study, RS5 was prepared from *Canna edulis* native starch and lauric acid through a hydrothermal method for the first time, and its nutritional intervention effects on hyperlipidemia in mice were investigated. The Canna edulis type 5 resistant starch (Ce-RS5) prepared using *Canna edulis* native starch and lauric acid exhibited a high compound index and resistant starch content, along with decreased swelling power and enhanced starch granule stability. The crystallinity of Ce-RS5 was decreased, and its crystal structure displayed a B+V pattern. Microscopically, the surface appeared rough with deepened grooves, and the granules were loose. Feeding mice with 1.5 g/kg and 3 g/kg of Ce-RS5 significantly reduced their body weight, positively regulated their blood lipid levels, and improved liver damage and fat accumulation. Additionally, Ce-RS5 promoted the abundance of beneficial gut bacteria, such as *norank_f_Muribaculaceae*, and inhibited the abundance of harmful bacteria like *Colidextribacter.* This study provides the first evidence of the hypolipidemic and weight loss effects of Ce-RS5 in hyperlipidemia mice.

## 1. Introduction

Chronic conditions, such as obesity and hyperlipidemia, are often instigated by the prolonged consumption of high-fat diets (HFDs), disrupting the lipid metabolic balance and posing a significant public health challenge [1]. Hyperlipidemia, particularly, stands out as a prominent issue associated with elevated cardiovascular disease risks [2]. Traditionally, therapeutic interventions for hyperlipidemia have heavily relied on pharmacological agents, yet multiple side effects and suboptimal patient adherence can plague long-term medication [3]. Consequently, the quest for safe and effective non-pharmacological therapies, particularly dietary modifications aimed at facilitating blood lipid profiles, has emerged as a pivotal research focus [4].

Starch is the main carbohydrate in the human diet and is one of the main sources of human physiological energy. In recent years, resistant starch (RS), a functional dietary component, has garnered substantial attention due to its unique physicochemical and physiological properties [5]. RS escapes digestion in the small intestine but undergoes fermentation by gut microbiota in the large intestine, producing short-chain fatty acids (SCFAs) that exhibit the potential to regulate lipid metabolism, improve insulin resistance, and alleviate inflammatory responses [6,7]. Among the various resistant starch (RS) types, RS5, a novel variant formed by the complexation of amylose with lipids, exhibits robust thermal stability and resistance to enzymatic degradation [8]. RS5 production, which eschews chemical reagents, is environmentally benign and straightforward, augmenting its appeal [9]. However, current research on RS5 remains fragmented, predominantly focused on its preparation methods, processing characteristics, and physicochemical properties, with limited exploration of its functional attributes, such as gut microbiota modulation or pharmacodynamic evaluations in disease models [10]. Tang et al. utilized an assisted ultrahigh-pressure homogenization method to synthesize a novel lotus root starch–myristic acid complex, demonstrating significant reductions in body weight, organ indices, fasting blood glucose, the effective modulation of blood lipid levels, the mitigation of liver injury, and enhanced colonic SCFAs in mice [11].

*Canna edulis*, an edible plant native to Guizhou, China, belongs to the genus Canna within the family Cannaceae [12]. Its rhizomes, rich in high-amylose starch, are recognized as an excellent raw material for RS5 production [13]. Zhang et al. reported weight loss and lipid-lowering effects attributed to *Canna edulis* type 3 resistant starch (Ce-RS3) [14]. Miu et al. found that Ce-RS3 can improve mild hyperlipidemia by regulating bile acid metabolism [15]. Li et al. observed that Ce-RS5 promotes the abundance of beneficial gut microbiota and generates abundant SCFAs during in vitro fermentation [16]. However, there is still no validation of Ce-RS5’s in vivo efficacy.

The purpose of this study was to prepare RS5 with *Canna edulis* native starch and lauric acid, observe its micro-morphology and physical and chemical characteristics, and explore its regulatory effects on blood lipids and microbiota in hyperlipidemia mice to provide theoretical and data support for the development of *Canna edulis* RS5 to be applied to the prevention and treatment of hyperlipidemia through functional food.

## 2. Materials and Methods

### 2.1. Materials

*Canna edulis* starch was purchased from Yilitai Co., Ltd. (Guizhou, China). Lauric acid and pullulanase were purchased from Maclin Co., Ltd. (Shanghai, China). Glucosidase and pancreatic alpha-amylase were purchased from Sigma Ltd. (Darmstadt, Germany) Simvastatin was acquired from Merck Investment Co., Ltd. (Saint Louis, MO, America). Total cholesterol (TC), triglyceride (TG), high-density lipoprotein cholesterol (HDL-C), low-density lipoprotein cholesterol (LDL-C), blood glucose (GLU), aspartate aminotransferase (AST), and alanine aminotransferase (ALT) kits were purchased from the Jianjieng Institute of Biology (Nanjing, China). A DNA extraction kit was purchased from Solaibao Technology Co., Ltd. (Beijing, China ) The other reagents were of analytical grade.

### 2.2. Preparation of Ce-RS5

According to Li et al. [16], the specific preparation process was as follows: An appropriate amount of *Canna edulis* (dry base) was mixed with distilled water to make 10% (*w*/*w*) starch milk, which was gelatinized at 95 °C for 20 min. Lauric acid was added to the starch milk at 10.0% (*w*/*w*, based on starch), and the sample was continuously stirred at 95 °C for 2 h. The obtained sample was cooled to room temperature. The sample was thoroughly washed with 50% ethanol, centrifuged at 1500× *g* for 10 min, and the supernatant was discarded. The samples were dried in a vacuum freeze dryer for 48 h, crushed and ground in a mortar, screened with 100 mesh, and stored in a sealed zip-lock bag for later use. In the following text, Ce-NS represents the *Canna edulis* native starch, and Ce-RS5 represents the *Canna edulis* native starch and lauric acid complex.

### 2.3. Structural Characterization

The structure of the Ce-NS and Ce-RS5 were observed by a ZEISS GeminiSEM 300 scanning electron microscope. The dried starch and RS5 particles were attached to the sample holder utilizing double-sided tape and coated with gold for 40 s using an Oxford Quorum SC7210 sputter coater. The operating voltage was set at 20 kV, and the starch shape was observed at different multiples [17].

### 2.4. XRD

The crystal structure of the sample was analyzed using a Japanese Rigaku Ultima IV X-ray diffractometer at a wavelength of 1.5418; the voltage was 40 kV, the current was 40 mA, the measurement angle was set to 2θ = 5–40°, and the scanning speed was 2°/min when the measurement was taken. The test target was a copper target. The resulting X-ray diffraction pattern assisted the Jade software in assembling the spike area and total area interval of the sample. The relative crystallinity (*RC*) of the sample to be measured could be obtained by integrating them separately. The formula is as follows:

$$RC\left(\%\right)=\frac{A_{c}}{A_{c}+A_{a}}\times 100\%$$

where *A_c_* represents part of the crystalline area and *A_a_* represents part of the amorphous area.

### 2.5. FTIR

Infrared spectroscopy was employed to ascertain the structural alterations in the starch post-processing. In a desiccated setting, 20 mg of the specimen was procured and thoroughly blended with an adequate quantity of anhydrous potassium bromide powder with a mortar. This mixture was thoroughly ground multiple times and then pressed onto a tablet press to form a transparent thin pellet. During the experimental process, the background spectrum was initially captured, followed by the acquisition of the infrared spectrum of the sample. The resolution was adjusted to 4 cm^−1^, with 32 scans conducted, and the wavenumber range was set within 400–4000 cm^−1^ for the test [18].

### 2.6. Swelling Power

A certain amount of starch was weighed and mixed with laboratory-grade deionized water to prepare a starch emulsion with a mass fraction of 10%. This mixture was then heated and stirred for 60 min in a water bath at varying temperatures (55 °C, 65 °C, 75 °C, 85 °C, and 95 °C). Following this, the emulsion was centrifuged at 3000 rpm for 10 min. The supernatant was discarded, and the combined mass of the centrifuge tube and the sediment was precisely determined [19]. The calculation formula for determining the relevant parameters is as follows:

$$SW(\%)=\frac{w_{p}}{w_{d}}$$

where *w_p_* refers to the mass of the expanded starch and *w_d_* refers to the mass of the starch on a dry basis.

### 2.7. Complex Index

The complex index was calculated according to Chen et al. [20]. A 100 mg sample was mixed in a 1 mL tube with 1 mL ethanol and 9 mL 1 M NaOH, dispersed in boiling water for 10 min, cooled, and diluted to 100 mL. A 5 mL aliquot was placed in another 100 mL flask; 1 mL of 1 M acetic acid and 1 mL iodine were added and diluted. After 10 min of color development, the absorbance was measured at 620 nm and repeated three times [21]. The calculation formula is as follows:

$$CI\left(\%\right)=\frac{A_{b}-A_{s}}{A_{b}}\times 100$$

where *A_b_* denotes the absorbance of the blank group and *A_s_* indicates the absorbance of the sample set.

### 2.8. In Vitro Digestibility

The resistant starch content was determined using a Megazyme Starch Resistance Assay Kit (K-RSAR). A total of 100 mg of the starch sample was precisely weighed and dried within a 10 mL centrifuge tube, and 6 mL of 0.1 M sodium acetate buffer and magneton was added. After vortex mixing, the sample was placed in a 37 °C magnetic stirring water bath for shock. The timer was started after adding 4 mL of a mixed enzymolysis solution of porcine pancrease and amyloglucosidase. After reacting for 2 h, 0.25 mL of the supernatant was mixed with 10 mL of 50% ethanol from each test tube to stop the enzymatic hydrolysis reaction. The centrifuge was run at 3000 r/min for 5 min, and 0.1 mL of the supernatant was mixed with 3 mL of GOPOD, and the light absorption value was measured at 510 nm [22]. The specific calculation method is as follows:

$$RS\left(\%\right)=\frac{\left(TG-G_{120}\right)\times 0.9}{TG}$$

where *G*_120_ is the glucose content produced after enzymatic hydrolysis for 2 h, and *TG* is the total glucose content of the sample.

### 2.9. Animals and Diets

Fifty-six-week-old male ICR mice, weighing 23 g ± 2 g, were acquired from Beijing Weitong Lihua Laboratory Animal Technology Co., Ltd. (Beijing, China; SCXK (Beijing) 2021-0011) and raised in the Laboratory Animal Center of Beijing University of Chinese Medicine, with room temperature maintained at 20–25 °C; humidity of 55–65%; light for 12 h, dark for 12 h; and a free diet during the experiment. The research was authorized by the Ethics Review Board of Beijing University of Chinese Medicine (No. BUCM-202307333-3042). Adaptive feeding was allowed for one week before the start of the trial. The mice were randomly allocated into a standard control group (NC, *n* = 10) and an HFD group (*n* = 40). The NC group received a standard diet, whereas the remaining group was administered an HFD consisting of 20% protein, 35% carbohydrates, and 45% fat. After eight weeks, the serum levels of the total cholesterol and triglycerides in the mice fed with the HFD were significantly higher than those in the control group, and the hyperlipidemia modeling was considered successful.

Then, the hyperlipidemia mice were randomly divided into four subgroups, the model group (MC), simvastatin group (Simvastatin, 0.01 g/kg), Ce-RS5 low-dose (Ce-RS5L, 1.5 g/kg) group, and Ce-RS5 high-dose (Ce-RS5H, 3 g/kg) group, for a 13 -week intervention. The mice in the NC and MC groups were given the same distilled water. At the end of the intervention, the mice were anesthetized, blood was taken from their orbits, and their sera were centrifuged at 4 °C and stored. Then the mice were dissected, and their livers and epididymal fat were removed and stored in 4% paraformaldehyde.

### 2.10. Biochemietry Analyses

The contents of TC, TG, LDL-C, HDL-C, AST, ALT, and GLU were determined by the corresponding kits. We strictly followed the instructions of the kits for specific operations (Nanjing Jiancheng Biological Technology Institute, China).

### 2.11. Pathological Histology Analyses

After fixation with 4.00% paraformaldehyde tissue fixative solution for 24 h, each liver was stained with hematoxylin–eosin (H&E) and oil red O, and the fat from the epididymis was stained with H&E, and the histopathological changes were observed and photographed under the microscope [23,24].

### 2.12. DNA Extraction

The DNA from the mice feces was extracted according to the kit, and the extracted genomic DNA was detected by 1% agarose gel electrophoresis.

### 2.13. Gut Microbiota Analysis

The DNA was qualified using universal primers 338F (5′-ACTCCTACGGGAGGCAGCA-3′) and 806R (5′-GGACTACHVGGGTWTCTAAT-3′); a PCR amplification was performed, and the PCR products were sequenced using the Illumina Mi Seq PE300 platform of Shanghai Meiji Biomedical Technology Co., Ltd. (Shanghai, China).

### 2.14. Statistical Analysis

All the experimental groups underwent at least three parallel procedures, and the experimental outcomes were presented as the mean ± SD. Using SPSS software, the pair data were analyzed, and a data comparison between multiple groups was performed using a one-way ANOVA for significance. The results were analyzed using the Turkey method and then compared using Origin 2021. GraphPad Prism 9.0 was used for the statistical analysis and graph generation.

## 3. Results

### 3.1. SEM

**Figure 1:**
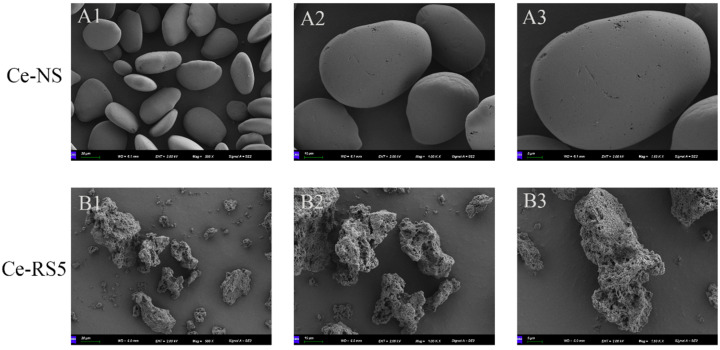
Scanning electron microphotographs of Ce-NS (**A1**–**A3**) and Ce-RS5 (**B1**–**B3**) at various magnifications (×500, ×1000, and ×1600).

The micro-morphology images of the Ce-NS and Ce-RS5 are shown in Figure 1. The Ce-NS has a smooth surface, appearing in an elliptical shape with a uniform distribution in the field of view. In contrast, the Ce-RS5 has lost its original state, exhibiting a relatively rough surface and an irregular flocculent structure. This observation is correlated with the gelatinization process of starch and the expansion of amylose during the RS5 formation phase [25].

### 3.2. XRD

**Figure 2:**
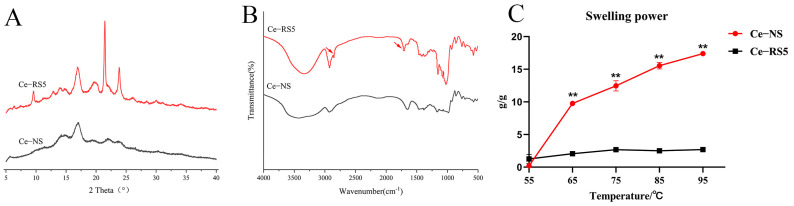
(**A**) XRD spectra, (**B**) FTIR pattern, and (**C**) swelling power of Ce-NS and Ce-RS5. Compared with the Ce-NS group, ** *p* < 0.01.

Figure 2A displays the XRD patterns of the Ce-NS and Ce-RS5. The Ce-NS exhibits distinct diffraction peaks at 5.7°, 15.2°, 17.1°, 22.1°, and 24.0°, indicating a typical B-type crystallinity [26]. However, the crystallinity of the Ce-RS5 undergoes changes, presenting with diffraction peaks at 5.5°, 7.5°, 12.9°, 16.9°, 19.8°, and 22.6°, indicative of a mixed B+V-type crystal structure [27]. The above results indicate that the formation of RS5 leads to a change in the crystal structure.

As Table 1 shows, Ce-RS5’s crystallinity was lower than Ce-NS’s. This observation can be attributed to the gradual penetration of water into the interstitial spaces of the starch microcrystals during the high-temperature gelatinization process. This leads to an extensive and irreversible water absorption and dissolution process within the starch granules, resulting in a linear degradation of the original crystalline structure.

### 3.3. FTIR

Compared to that of the Ce-NS, the FTIR spectrum of the Ce-RS5 did not exhibit significant changes in its peak shapes. Still, two new absorption peaks emerged near 2850 cm^−1^ and 1710 cm^−1^ (Figure 2B). Specifically, the peak observed at 2850 cm^−1^ was attributed to the stretching vibrations of the methyl and methylene groups in fatty acids. Additionally, the absorption peak at 1710 cm^−1^ indicated the stretching vibrations of the carbonyl groups within fatty acids, serving as a distinctive marker for the formation of RS5 [28,29].

Table 1 summarizes the characteristic absorption peaks of the deconvoluted infrared spectra for the Ce-NS and Ce-RS5. The ratio IR_1047/1022_ reflects the orderliness of the starch crystals, whereas IR_995/1022_ indicates the amorphous region of the starch molecules [29]. The Ce-RS5 exhibits an increased IR995/1022 ratio compared to Ce-NS, though the difference is not statistically significant. However, a substantial decrease in IR_1047/1022_ is observed for the Ce-RS5, which is attributed to the disruption of the double-helical structures in the crystalline regions of starch by high-temperature heating, leading to a decrease in the orderliness of the starch molecules [30]. This finding is consistent with the crystallinity results.

### 3.4. Swelling Power

The swelling capacity can serve as a metric to assess the extent of interaction among starch chains, mirroring the interconnectivity between the amorphous and crystalline domains [31]. Compared to the Ce-NS, the incorporation of lauric acid significantly reduced the swelling power of the starch granules at various temperatures (Figure 2C). This reduction can be attributed to the influence of the Ce-RS5 formation on the hydrophilic hydroxyl structure of amylose, inhibiting the permeation of water molecules into the interior of the starch granules and their subsequent binding [32]. Consequently, the rate of water uptake and expansion of the starch granules was moderated to some degree.

### 3.5. Complex Index (CI)

As shown in Table 2, the complex index of the Ce-RS5 is presented. The helical hydrophobic cavity structure of amylose can form complexes with exogenous lipids through hydrophobic interactions [33]. The complexation index indicates the degree of compounding between starch and fatty acids [34]. The larger the complex index, the weaker the ability of amylose to bind to iodine, and the higher the degree of complexity with lauric acid. After multiple repetitions of the measurements, the results indicate that the compound yield between Canna starch and lauric acid is relatively high and stable, with a value of 57.11%.

### 3.6. In Vitro Digestibility

As detailed in Table 2, the RS content of the Ce-NS and Ce-RS5 are presented. The Ce-NS exhibits a high degree of resistance to enzymatic hydrolysis, with an RS content of 53.2%. For the Ce-RS5, the incorporation of lauric acid during its preparation process contributes to a certain extent to the reduction in the recrystallization of the starch single helices. This leads to enhanced resistance to enzymatic degradation [35], rendering the starch–lauric acid complex a promising material with broader application prospects in the food industry and other relevant fields.

### 3.7. Changes in Body Weight, Lee’s Index, and eWAT Weight

**Figure 3:**
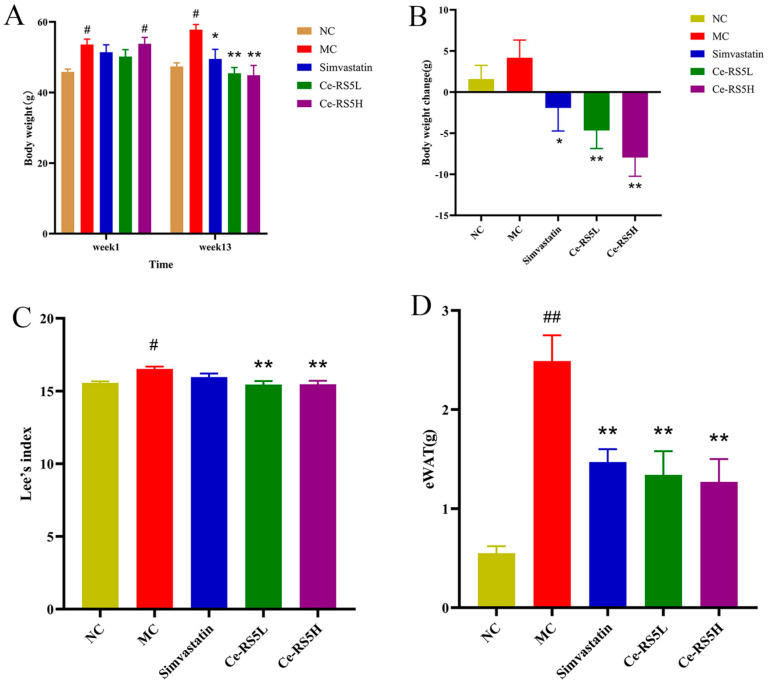
(**A**) Body weight, (**B**) body weight change, (**C**) Lee’s index, and (**D**) eWAT of different groups. Compared with the NC group, # *p* < 0.05, ## *p* < 0.01; compared with the MC group, * *p* < 0.05, ** *p* < 0.01; the data show mean ± SD value, with n = 8 mice per group.

Figure 3A depicts the post-modeling body weight variations, wherein the HFD groups exhibit significantly greater weights than the NC group in week 1, suggesting a successful model induction. After 13 weeks of the nutritional intervention, the mice in the MC group still have a significantly higher body weight than those in the NC group due to the continuous HFD. However, the intervention with simvastatin and Ce-RS5 may alleviate this phenomenon, showing a trend of weight loss, and the Ce-RS5H has a better effect with a dose-dependence (Figure 3B). Specifically, the weight with the Ce-RS5L was reduced by 9.4% and that with the Ce-RS5H by 16.5% compared to the initial weights. These findings suggested that Ce-RS5 has potential weight loss effects.

Lee’s index can be utilized to assess the degree of obesity in mice [36]. The 13-week HFD resulted in a significantly higher Lee’s index in the MC group compared to the NC group. In contrast, the simvastatin, Ce-RS5L, and Ce-RS5H groups showed a decreasing trend, with significant differences observed for the low and high doses of Ce-RS5 (Figure 3C).

Figure 3D shows the weight of the epididymal fat in the mice from each group. The MC group displays a significant increase in epididymal fat mass compared to the NC group. However, Ce-RS5 demonstrates a comparable trend to that of the positive drug, markedly reducing epididymal fat accumulation resulting from long-term HFD consumption. In summary, the above results again confirm the good lipid-lowering effect of Ce-RS5.

### 3.8. The Lipid Content in the Serum

**Figure 4:**
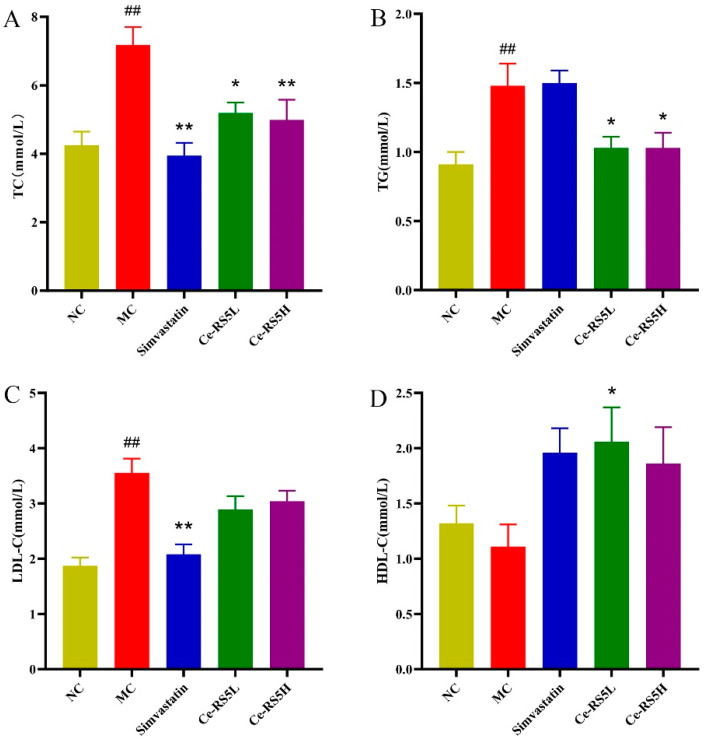
(**A**) TC, (**B**) TG, (**C**) LDL-C, and (**D**) HDL-C of different groups. Compared with control group, ## *p* < 0.01; compared with model group, * *p* < 0.05, ** *p* < 0.01; data show mean ± SD value, with n = 8 mice per group.

Due to the long-term HFD intake, the mice in the MC group exhibited abnormal blood lipids, with the serum TC, TG, and LDL-C showing a significant upward trend. The long-term intake of foods with a high-fat content increases the rate of fat decomposition, inhibits the synthesis of lipids, produces a large number of fatty acids, and improves the triglyceride (TG) and TC content in serum. Conversely, Ce-RS5 can regulate dyslipidemia in hyperlipidemia mice by decreasing serum TC, TG, and LDL-C, and increasing HDL-C (Figure 4A–D). Li et al. found that a ginkgo starch and lauric acid complex can reduce body weight and lower serum TG and TC in hyperlipidemic rats [37]. The primary mechanism by which RS regulates lipids is by reducing the amount of dietary cholesterol absorption and promoting the excretion process of cholesterol, thus effectively decreasing plasma cholesterol [38]. These results indicate that Ce-RS5 is a starch with good biological activity and can be used as a nutritional intervention strategy to alleviate hyperlipidemia.

### 3.9. Liver Function and Glucose Content

**Figure 5:**
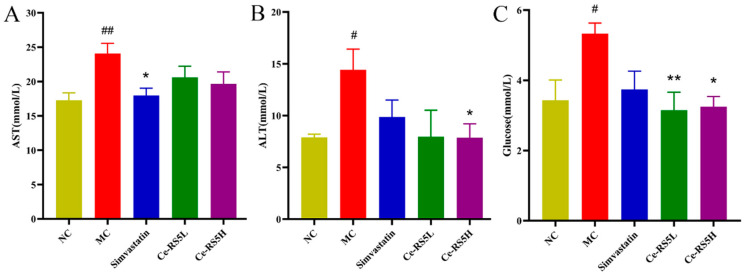
(**A**) AST, (**B**) ALT, and (**C**) glucose of different groups. Compared with control group, # *p* < 0.05, ## *p* < 0.01; compared with model group, * *p* < 0.05, ** *p* < 0.01; data show the mean ± SD value, with n = 8 mice per group.

As depicted in Figure 5A,B, the AST and ALT in the MC group mice are significantly increased. Under the influence of an HFD, hepatic lipid synthesis increases and gradually accumulates in the hepatocytes. The excessive deposition of lipids into the hepatocytes may lead to incomplete hepatocyte membrane structures, thereby impairing the normal function of the hepatocytes themselves, resulting in hepatocyte damage and the production of AST and ALT [39]. Simvastatin can reduce the AST and ALT and reverse liver damage. It is observed that Ce-RS5 has a similar effect to simvastatin, with a significantly reduced AST and ALT. These results indicate that Ce-RS5 exerts a favorable regulatory and protective effect against high-fat-induced liver dysfunction.

Furthermore, Figure 5C shows that Ce-RS5 significantly ameliorates the elevation in serum glucose in the MC group of mice induced by the long-term HFD. RS is an excellent substrate for producing SCFAs in the gut [40]. Studies have suggested that the beneficial activities of SCFAs include reducing serum glucose and improving inflammatory states [41].

### 3.10. Pathological Analysis of Liver and eWAT

**Figure 6:**
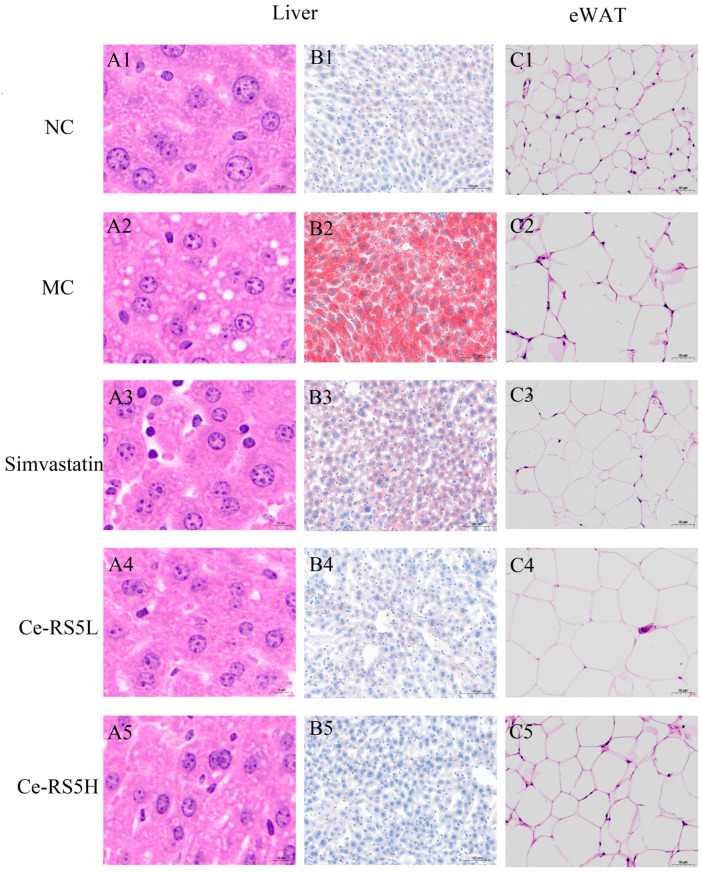
HE staining and oil red O staining of liver and HE staining of epididymal fat of different groups (×25, ×25, and ×100). (**A1**–**A5**) liver; (**B1**–**B5**) liver; (**C1**–**C5**) epididymal fat.

The HE staining of the mice liver sections revealed that the NC group of mice exhibited a typical hepatocyte structure. In contrast, the MC group of mice showed significantly enlarged hepatocytes filled with numerous lipid vacuoles. The liver is one of the most vital metabolic organs in the body, primarily responsible for lipid synthesis and storage [42]. When excessive lipid deposition occurs in the liver, subsequent β-oxidation may trigger inflammatory responses and oxidative stress [43]. Notably, the Ce-RS5 intervention significantly reduced the number of lipid vacuoles and mitigated the degree of hepatocyte deformation (Figure 6A1–A5). Oil Red O staining was used to directly visualize the differential changes in the hepatic lipid content among the groups. Oil red O can dye lipid droplets red, while other areas are blue or dark blue [44]. Compared to the NC group, the MC group of mice displayed abundant red lipid droplets indicative of diffuse fatty degeneration, markedly improved by the Ce-RS5 intervention, consistent with the HE staining results (Figure 6B1–B5). He et al. found that lotus seed RS can alleviate liver steatosis caused by an HFD [45].

The HE staining results of the mouse epididymal fat showed that the fat cells in the NC group were smaller and evenly arranged. In comparison, the fat cells in the MC group were significantly larger, irregular in shape, and significantly reduced in number. Simvastatin and Ce-RS5 can significantly alleviate adipocyte damage in hyperlipidemic mice (Figure 6C1–C5). These results suggest that Ce-RS5 ameliorates HFD-induced liver injury and fat accumulation in mice.

### 3.11. Changes in Intestinal Microbiota

The research has indicated that the emergence of metabolic disorders, including hyperlipidemia, is intimately linked to alterations in the gut microbiota [46]. Based on the above pharmacology, the gut microbiota from the mouse feces was analyzed.

**Figure 7:**
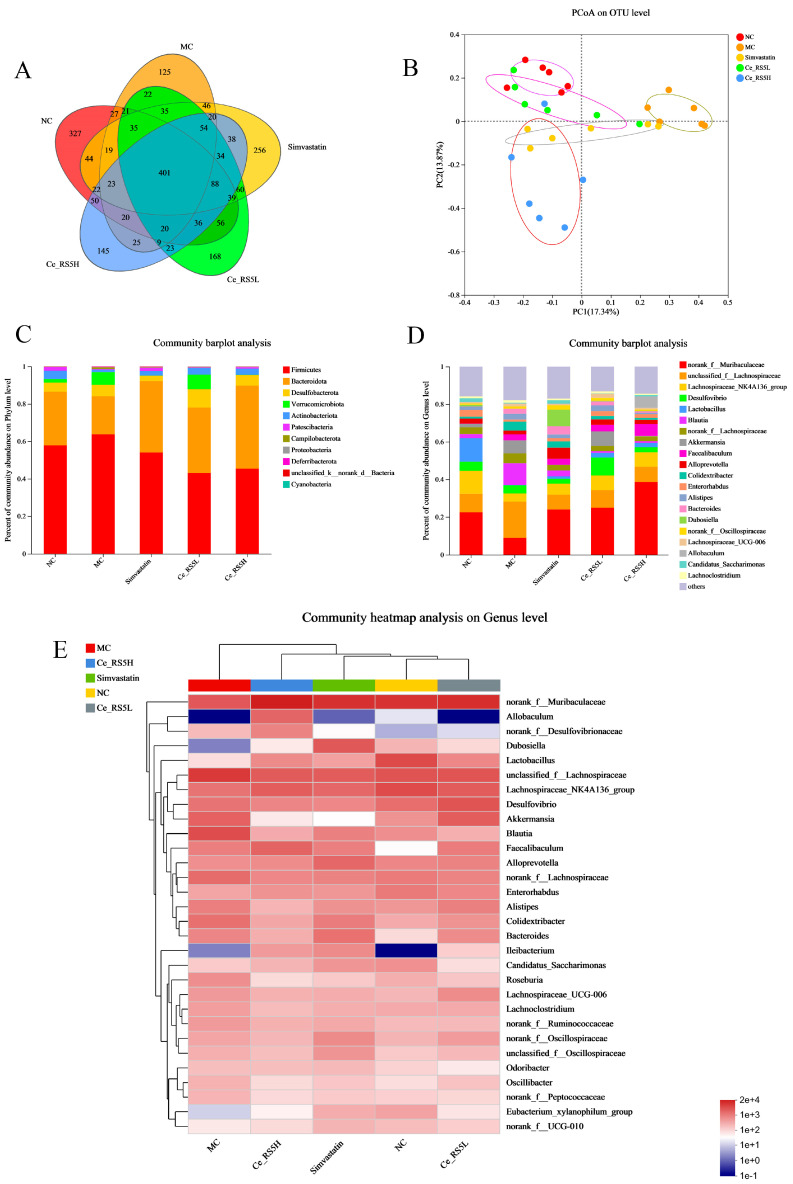
(**A**) Venn, (**B**) PCoA, (**C**) phylum, (**D**) genus, and (**E**) heatmap of different groups. D data show mean ± SD value, with n = 6 mice per group.

High-throughput sequencing was conducted on the V3–V4 region of the 16S rRNA gene extracted from the fecal samples of the mice in each group, utilizing the Illumina sequencing platform. A Venn diagram effectively reflects the similarity and overlap in the species composition among the samples (Figure 7A) [47]. The results identify that 401 OTUs are shared among all five groups, with the NC group having the highest number of OTUs (327) and the MC group the lowest (125).

To clarify the changes in the microbial communities within the feces of the mice from the various groups, 16S rRNA gene sequencing was adopted. The α-diversity indices are displayed in Table 3. Notably, the Chao1 and ACE indices are positively connected with community richness, while the Shannon and Simpson indices are positively associated with community diversity [48]. The alpha-diversity index of the MC group is decreased significantly compared to the NC group, while after the Ce-RS5 intervention, the Chao1 and ACE indices are increased, while the Shannon index is decreased. This indicates that Ce-RS5 can enhance gut microbiota richness in hyperlipidemic mice, but does not affect its diversity.

A PCoA plot was employed to gain insight into the overall composition of the microbial communities (Figure 7B) [49]. The two primary coordinates (PC1: 17.34%; PC2: 13.87%) accounted for 31.21% of the total variability observed across the samples. The clear separation between the NC and MC groups on the PCoA plot indicates a significant change in microbial diversity after 13 weeks of the HFD feeding. The samples from the simvastatin, Ce-RS5L, and Ce-RS5H groups, while distinct from the MC group, tend towards the NC group, suggesting similarity within the groups and differences between the groups, with the Ce-RS5H group farthest from the MC group. This indicates that the Ce-RS5 intervention alters the beta diversity of the mice’s intestinal microbiome.

Figure 7C displays the relative abundance of the microbial phyla in the mice. The total microbial abundance is predominantly composed of Firmicutes and Bacteroidetes, accounting for over 80% of the population. The MC group fed an HFD for an extended period shows an increased level of Firmicutes and decreased Bacteroidetes, while the opposite trend is observed after the Ce-RS5 intervention. An increase in Firmicutes is positively correlated with fat storage, whereas an increase in Bacteroidetes is associated with fat loss [50]. These alterations in abundance lead to a notable reduction in the Firmicutes-to-Bacteroidetes (F/B) ratio, as reflected in Table 3. Studies have indicated that an elevated F/B ratio is correlated with an increased risk of obesity, aligning with the weight loss outcomes observed in the mice subjected to the Ce-RS5 intervention in the present study [51].

Figure 7D shows the microbial composition of each group of mice at the genus level. Compared to the NC group, the MC group exhibits a decrease in norank_f_Muribaculaceae and Lachnospiraceae_NK4A136_group and an increase in *unclassified_f_Lachnospiraceae*, *Blautia*, *norank_f_Lachnospiraceae*, and *Colidextribacter*. However, after the Ce-RS5 intervention, these trends reverse, approaching that observed in the NC group (Figure 7D). Among them, the undetermined genus (*norank_f_Muribaculaceae*) of the family Murine bacterae, a class of beneficial bacteria that produces SCFAs, is significantly negatively correlated with obesity-related indicators [52]. The decreased abundance of *Colidextribacter* in Ce-RS5 has been reported as a harmful bacterial genus associated with obesity [53].

Figure 7E displays a heatmap of the microbial communities with the highest abundance, being in the top 30 at the genus level. Ce-RS5 enriched the abundance of bacteria, such as *norank_f_Muribaculaceae*, *unclassified_f_Lachnospiraceae*, *Lachnospiraceae_NK4A136_group*, and *Faecalibaculum*. Yuan’s research indicated that *norank_f_Muribaculaceae* negatively correlates with the total triglycerides and total cholesterol in epididymal fat, perirenal fat, and the liver [54]. This is confirmed by the increase in *norank_f_Suribaculaceae* and the improvement in dyslipidemia in the Ce-RS5 group in this study. The *Lachnospiraceae NK4A136_group* has been implicated in generating SCFAs, exhibiting the potential to regulate glucose and lipid metabolism while alleviating inflammatory responses [55]. Yang et al. reported that resistant starch could enhance the prevalence of beneficial bacteria, such as Faecalibaculum, and exhibit a recuperative effect on hepatic steatosis and intestinal dysbiosis induced by an HFD, findings that are consonant with the outcomes of this investigation [56].

In summary, our findings suggest that Ce-RS5 may improve hyperlipidemia by regulating the abundance of gut microbiota.

## 4. Conclusions

In summary, type 5 RS was successfully prepared by a hydroheat treatment using *Canna edulis* native starch and lauric acid as the raw materials. Ce-RS5 showed an apparent B+V-shaped crystal structure; the composite index was 57.11%; the RS content was 56%; and the microstructure of Ce-RS5 was significantly changed compared with Ce-NS’s, from smooth to irregular and rough. In addition, Ce-RS5 exhibited significant weight loss and lipid-lowering effects in hyperlipidemic mice. The daily intake of a specific dose of Ce-RS5 significantly reduced the mice’s weight gain and fat gain caused by a long-term high-fat diet, effectively regulated the lipid levels of the model mice, reduced fat accumulation, and alleviated liver inflammation by reducing AST and ALT levels. Simultaneously, a 16S rRNA gene sequencing analysis revealed that the Ce-RS5 intervention significantly enriched the abundance of beneficial bacterial genera such as *norank_f_Muribaculaceae,* and reduced obesity-associated harmful bacterial genera like *Colidextribacter*. The F/B value was significantly reduced, which also confirmed that Ce-RS5 could improve obesity and metabolic disorders. This study helps us understand the probiotic function and hypolipidemia effect of Ce-RS5.

Starch, as a carbohydrate, accounts for a high proportion of humans’ daily dietary intake, suggesting that Ce-RS5 could be used in the diet to prevent and treat hyperlipidemia. This study provides a theoretical reference and data support for developing functional foods based on *Canna edulis* native starch or related functional products with preventive and therapeutic effects for hyperlipidemia patients.

In the future, the possible mechanisms for the regulating of lipid metabolism could be analyzed from the perspective of inflammatory pathways, fatty acids, and other metabolites.

## Figures and Tables

**Table 1:** Crystallinity and infrared (IR) ratios of Ce-NS and Ce-RS5.

Sample	Crystallinity (%)	IR Ratio of 1047/1022 cm^−1^	IR Ratio of 995/1022 cm^−1^
Ce-NS	36.68 ± 2.02 a	1.02 ± 0.12 a	0.98 ± 0.01 a
Ce-RS5	29.22 ± 0.98 b	0.87 ± 0.01 b	1.01 ± 0.01 a

Values presented are mean ± SD. Values within same column followed by different letters denote statistically significant differences at *p* < 0.05.

**Table 2:** CI and RS content of Ce-NS and Ce-RS5.

Sample	CI (%)	RS (%)
Ce-NS	/	53.20 ± 2.27
Ce-RS5	57.11 ± 0.22	56.12 ± 0.82

Values presented are mean ± SD.

**Table 3:** The α-diversity index of microbial communities of different groups.

Group	α-Diversity Index				F/B
	Chao1	ACE	Shannon	Simpson	
NC	636.27 ± 49.98 a	645.18 ± 50.48 a	4.21 ± 0.23 a	0.04 ± 0.02 b	1.09 ± 0.20 a
MC	507.19 ± 34.10 b	515.42 ± 35.52 b	3.88 ± 0.09 ab	0.05 ± 0.01 ab	3.79 ± 0.48 b
Simvastatin	605.89 ± 56.87 ab	624.35 ± 26.68 ab	4.12 ± 0.11 a	0.04 ± 0.01 b	2.36 ± 0.62 b
Ce-RS5L	564.96 ± 33.77 ab	568.12 ± 29.90 ab	3.41 ± 0.13 ab	0.06 ± 0.01 ab	1.45 ± 0.31 a
Ce-RS5H	516.14 ± 17.50 b	522.76 ± 34.54 b	3.51 ± 0.23 b	0.10 ± 0.03 a	1.86 ± 0.89 a

Values presented are mean ± SD. Values within same column carrying different letters denote statistically significant differences at *p* < 0.05.

## Data Availability

The data presented in this study are available on request from the corresponding author. The data are not publicly available due to privacy restrictions.
